# Adiposity Reduction by *Cucumis melo* var. *gaettongchamoe* Extract in High-Fat Diet-Induced Obese Mice

**DOI:** 10.3390/nu15153292

**Published:** 2023-07-25

**Authors:** Sun Young Park, Ji Eun Kim, He Mi Kang, Hee Jin Song, Nam Jun Kang, Dae Youn Hwang, Young-Whan Choi

**Affiliations:** 1 Bio-IT Fusion Technology Research Institute, Pusan National University, Busan 46241, Republic of Korea; sundeng99@pusan.ac.kr; 2 Department of Biomaterials Science, Pusan National University, Miryang 50463, Republic of Korea; prettyjiunx@naver.com (J.E.K.); hejin1544@naver.com (H.J.S.); dyhwang@pusan.ac.kr (D.Y.H.); 3 Department of Horticultural Bioscience, Pusan National University, Miryang 50463, Republic of Korea; mimi2965@naver.com; 4 Department of Horticulture, Gyeongsang National University, Jinju 52828, Republic of Korea; k284077@gnu.ac.kr

**Keywords:** *Cucumis melo* var. *gaettongchamoe*, anti-obesity, high-fat diet (HFD), hepatic steatosis

## Abstract

This study investigated the anti-obesity effects of *Cucumis melo* var. *gaettongchamoe* (CG) in mice fed a high-fat diet (HFD). The mice received CG water extract (CGWE) treatment for 8 weeks, and changes in body weight and serum lipid levels were analyzed. The HFD + vehicle group showed a significant increase in body weight compared to the control group, while the HFD + CGWE and HFD + positive (orlistat) groups exhibited reduced body weight. Lipid profile analysis revealed lower levels of total cholesterol, triglycerides, high-density lipoprotein, and low-density lipoprotein cholesterol in the HFD + CGWE group compared to the HFD + vehicle group. The HFD + vehicle group had increased abdominal fat weight and fat content, whereas both HFD + CGWE groups showed significant reductions in abdominal fat content and adipocyte size. Additionally, CGWE administration downregulated mRNA expression of key proteins involved in neutral lipid metabolism. CGWE also promoted hepatic lipolysis, reducing lipid droplet accumulation in hepatic tissue and altering neutral lipid metabolism protein expression. Furthermore, CGWE treatment reduced inflammatory mediators and suppressed the activation of the mitogen-activated protein kinase pathway in hepatic tissue. In conclusion, CGWE shows promise as a therapeutic intervention for obesity and associated metabolic dysregulation, including alterations in body weight, serum lipid profiles, adipose tissue accumulation, hepatic lipolysis, and the inflammatory response. CGWE may serve as a potential natural anti-obesity agent.

## 1. Introduction

Obesity is a global epidemic associated with various health problems, including type 2 diabetes, dyslipidemia, and fatty liver disease. It is characterized by an increase in both the number (hyperplasia) and size (hypertrophy) of fat cells, leading to an increase in adipose tissue mass. Obesity is linked to a range of diseases, such as type 2 diabetes, hypertension, dyslipidemia, fatty liver, gallbladder disease, myocardial infarction, stroke, gout, osteoarthritis, colorectal cancer, breast cancer, and mental disorders [1,2,3]. Adipose tissue serves as a biological calorie reservoir, expanding in response to excess nutrients and releasing lipids during energy deficiency. However, excessive fat accumulation and dysfunctional adipocytes lead to alterations in plasma lipid and lipoprotein levels, including augmented low-density lipoprotein (LDL) cholesterol levels and, triglyceride (TG) and decreased high-density lipoprotein (HDL) cholesterol levels, contributing to the pathogenesis of obesity-related disorders [4,5,6].

Maintaining an appropriate balance between energy storage and expenditure is crucial for preventing and treating obesity. The number of adipocytes remains relatively stable throughout adulthood. Recent evidence suggests that prolonged high-fat diet (HFD) consumption preferentially initiates adipogenesis in the white adipose tissue, indicating its involvement in obesity and related chronic diseases [7,8,9]. The development of white adipose tissue is regulated by various transcription factors, including CCAAT/enhancer-binding protein α (C/EBPα) and peroxisome proliferator-activated receptor γ (PPARγ). These transcription factors performance essential roles in lipid metabolism and gene regulation related to adipogenesis and adipocyte function, including the expression of fatty acid synthase (FAS), adipocyte fatty acid-binding protein-2 (aP2), and lipoprotein lipase [10,11,12]. Dysregulation of these genes is associated with adipocyte dysfunction and insulin resistance in obesity. Therefore, targeting adipogenesis and triglyceride synthesis holds promise for regulating adipose tissue growth and addressing various metabolic disorders in obesity [13,14,15].

*Cucumis melo* var. *gaettongchamoe* (CG) is a variant of watermelon belonging to the Cucurbitaceae family, with origins in African and Asian Indian regions [16]. Guo et al. (2022) reported the anti-obesity effect of vitalmelon, which was developed by hybridization of Korean wild melon (CG) crossed with Moneymaker melon cultivar (*Cucumis melo* var. *makuwa* Makino). The Korean native CG used in this study is a seed-parent (female) plant, which is different from the F1 hybrid of vitalmelon (*Cucumis melo* var. *vitalmelon*). Watermelon, widely cultivated in South Korea, China, and Japan, is renowned for its sweet taste and unique aroma, making it a popular summer fruit [17]. It contains high sugar content, abundant carbohydrates, carotene, and vitamins A and C, along with phosphorus and other bioactive compounds [18]. Besides its hydrating properties due to its high water content of over 90%, watermelon is also recognized for its diuretic effects and traditional uses in Korean medicine for conditions such as fever, constipation, jaundice, and gastrointestinal disorders [19]. Furthermore, watermelon has been investigated for its beneficial effects in acute gastrointestinal diseases and strokes, with reported anti-inflammatory, analgesic, antidiabetic, anti-angiogenic, and anti-ulcer properties [20] Additionally, certain components of watermelon, such as cucurbitacins B and E, volatile aromatic compounds, phenolic compounds, and flavonoids, have been associated with various physiological activities, including potential anti-proliferative effects on cancer cells, suggesting their possible application in cancer treatment and prevention [21,22]. Intriguingly, *Cucumis melo* var. *gaettongchamoe* (CG) stands out as a distinctive type of watermelon, spontaneously growing from discarded seeds after consumption. Although small and seemingly tasteless, CG watermelon has found practical use in medicinal contexts [23,24].

In this study, we aimed to investigate the effects of CG water extract (CGWE) on the body weight, adipose tissue mass, and metabolic parameters of HFD-fed mice. Through comprehensive in vitro and in vivo experiments, we sought to provide detailed insights into the potential therapeutic benefits of CGWE in combating adipogenesis and obesity.

## 2. Materials and Methods

### 2.1. CG Collection and Extract Preparation

CG plants were collected from Jinju, Korea, and cultivated at the Department of Horticulture, Faculty of Agriculture, Gyeongsang National University. To ensure stable characteristics, the collected CG plants underwent six rounds of backcrossing. In late spring 2019, the CG plants were grown, and the fruits were harvested at their ripe stage. The CG fruits were then dried using a freeze dryer (Ilshin, Dongducheon, Korea), ground into a fine powder, and passed through a 30-mesh sieve. The powdered sample was stored at −20 °C until further extraction. Prior to the experiment, 10 g of the powdered sample was retrieved and extracted using a sonicator (JAC Ultrasonic, Seoul, Korea) with ten times the volume of distilled water (*w/v*) at room temperature for 1 h. The resulting extract was filtered through Whatman No. 2 filter paper (Whatman International Ltd., Maidstone, England, UK) and then dried with a rotary evaporator (Heidolph, Heidolph Korea Ltd., Seoul, Republic of Korea), resulting in 4.3 g of CG extract. The obtained CG extract was stored in a glass bottle and kept at −20 °C until use. For this study, the aqueous extracts of *Cucumis melo* var. *gaettongchamoe* were labeled as CGWE.

### 2.2. Animal Study

Ethical guidelines were strictly followed, and the Pusan National University Institutional Animal Care and Use Committee (PNU-IACUC) approved all animal experimental procedures under approval code (PNU-2022-0139) and approval data (17 March 2022). Forty male C57BL/6 mice, aged 7 weeks, were obtained from Samtako Bio-Korea, Inc. (Osan, Republic of Korea) and housed at the Pusan National University Laboratory Animal Resources Center. The center holds accreditations from the Korea Food and Drug Administration (KFDA) (Accredited Unit Number-000231) and the Association for Assessment and Accreditation of Laboratory Animal Care (AAALAC) International (Accredited Unit Number 001525). The mice were divided into two groups: a non-HFD group (*n* = 8) and an HFD-fed group (*n* = 32). The non-HFD group received a diet containing 10% kcal fat, while the HFD-fed group received a high-fat diet (HFD) containing 60% kcal fat from Research Diets. The HFD group was further divided into three subgroups: HFD + Po, HFD + L, and HFD + H. The HFD + Po group received a daily oral dose of 10 mg/kg body weight orlistat, while the HFD + L and HFD + H groups received oral doses of 1 and 10 mg/kg body weight CGWE, respectively. The No and HFD + Ve groups were administered the same volume of distilled water orally as controls. Throughout the study, the mice were maintained in specific pathogen-free conditions with a strict light cycle (on at 08:00 h; off at 20:00 h), at a temperature of 23 ± 2 °C, and relative humidity of 50 ± 10%. They had ad libitum access to a standard irradiated chow diet (Samtako BioKorea Inc., Osan, Republic of Korea) and water. Body weight was measured twice a week using an electronic balance, and food intake and water consumption were measured weekly following KFDA guidelines. Upon completing the experiment, the mice were euthanized via CO_2_ gas, and various tissue samples were collected and stored in Eppendorf tubes at −70 °C for further analysis.

### 2.3. HPLC Analysis

To determine the content of cucurbitacin A and B in CGWE extracts, an HPLC system consisting of an Agilent 1100 system (Santa Clara, CA, USA), an auto-injector, and a column temperature regulator was used. A reversed-phase analytical Luna C18 column (150 mm × 4.6 mm I.d., 5 μm particle diameter; Pheomenex, Torrance, CA, USA) was employed for the separation. The mobile phases comprised 0.01% formic acid in water (mobile phase A) and acetonitrile (mobile phase B). The analysis involved a gradient elution: 40% B at 0 min, 40% B from 0 to 30 min, and 100% B from 30 to 40 min. Each sample was injected with a volume of 20 μL. The flow rate was maintained at 0.5 mL/min, and the column temperature was kept constant at 30 °C during the analyses.

### 2.4. Serum Biochemical Analysis

After the completion of the final treatment, all C57BL/6 mice were subjected to an 8-hour fasting period. Subsequently, euthanasia was carried out by administering CO_2_ gas, and whole blood samples were collected from the abdominal veins using a 1-mL syringe and needle. Serum for biochemical analyses was obtained by centrifuging the collected blood samples at 1500× *g* for 15 min. The levels of several serum biochemical components, including total cholesterol (TC), triglycerides (TG), high-density lipoprotein (HDL), and low-density lipoprotein (LDL), were measured using an automatic serum analyzer (Hitachi 747; Hitachi, Tokyo, Japan). All assays were conducted twice, using fresh serum samples.

### 2.5. Histopathological Analysis

Liver and adipose tissue samples retrieved from the mice were fixed by immersion in a 10% formalin solution for 48 h. Subsequently, the fixed samples were embedded in paraffin wax and cut into 4 μm-thick sections. These sections were then subjected to H&E staining using Sigma-Aldrich reagents (Saint Louis, MI, USA). An optical microscope (Leica Microsystems, Wetzlar, Germany) was employed to analyze the adipocyte size and lipid droplet number in both the adipose tissue and liver sections.

### 2.6. Quantitative Real Time–Polymerase Chain Reaction (qRT-PCR) Analysis

Total RNA was extracted from frozen mid-colon tissues using the RNA Bee solution (Tet-Test Inc., Friendswood, TX, USA). The RNA concentration was determined, and cDNA was synthesized from the RNA using a mixture of dNTPs, and reverse transcriptase (Superscript II, Thermo Fisher Scientific Inc.), oligo-dT primers (Thermo Fisher Scientific Inc., Waltham, MA, USA). For real-time quantitative PCR (RT-qPCR), the cDNA template was combined with 2× Power SYBR Green (Toyobo Co., Osaka, Japan). The primer sequences employed for mRNA analysis are outlined in Table 1.

### 2.7. Western Blotting Analysis

Liver proteins were extracted using Pro-Prep Protein Extraction Solution (Intron Biotechnology Inc., Seongnam, Republic of Korea) following the manufacturer’s instructions. The protein concentration was determined with the SMARTTM Bicinchoninic Acid Protein assay kit (Thermo Fisher Scientific Inc., Wilmington, MA, USA). Next, 30 μg of protein was separated on a 4–20% SDS-PAGE gel, transferred onto nitrocellulose membranes, and blocked with 5% nonfat milk in Tris-buffered saline with Tween 20 (TBST). The membranes were then incubated overnight at 4 °C with specific primary antibodies. After washing with TBST, the membranes were incubated with horseradish peroxidase-conjugated secondary antibodies. Protein bands were visualized using a chemiluminescence kit, and images were captured using a digital camera equipped with the FluorChem^®^ FC2 Imaging system. Protein densities were analyzed using the AlphaView Program, version 3.2.2.

### 2.8. Statistical Analysis

Statistical analyses were conducted using SPSS software version 10.10 (SPSS, Inc. Chicago, IL, USA). One-way analysis of variance (ANOVA) followed by Tukey’s post hoc test was utilized for data with a normal distribution. All values are expressed as means ± standard deviations. A *p*-value less than 0.05 was considered statistically significant, indicating meaningful differences among the experimental groups.

## 3. Results

### 3.1. Effects of CGWE Treatment on Body Weight and Serum Lipid Profiles in HFD-Induced Obese Mice

**Figure 1:**
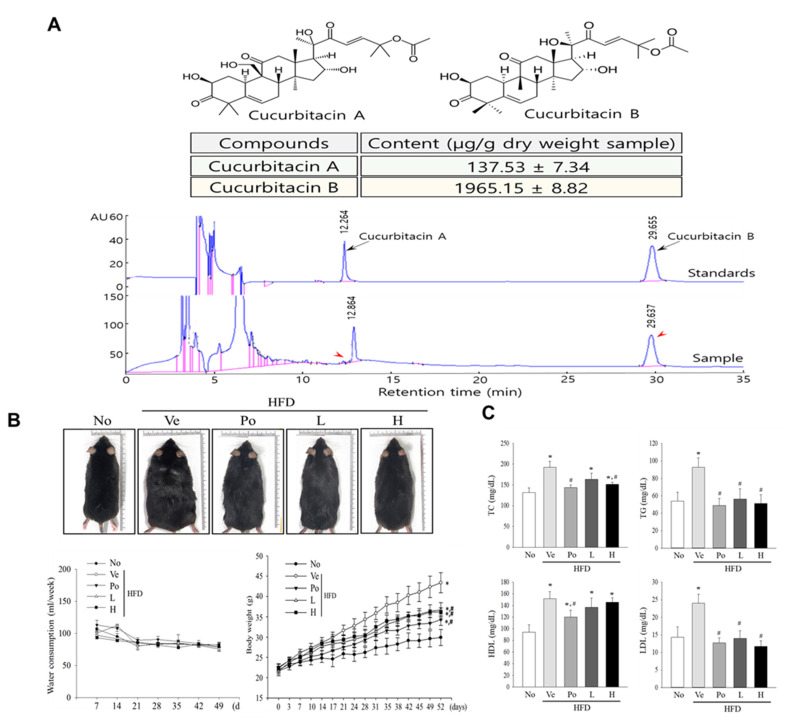
Effects of vehicle, Po (orlistat), and CGWE on body weight and serum lipid profile in HFD-induced obese C57BL/6 mice. (**A**) HPLC chromatogram displaying the content of cucurbitacin A and B in dried CGWE fruit samples. Separation was performed using a Luna C18 column with 0.01% formic acid in water and acetonitrile as mobile phases. (**B**) Comparison of water consumption and body weight changes among the experimental groups over 7 weeks. The percentage body weight gain from 0 to 7 weeks was calculated. (**C**) Measurement of TC, TG, HDL, and LDL-C in the serum of each group. * *p* < 0.05 vs. the No group; # *p* < 0.05 vs. the HFD + Ve-treated group.

In this study, we aimed to investigate the potential anti-obesity effects of CGWE in mice fed a high-fat diet. Cucurbitacins, such as A, B, E, and I, found in various variants of the Cucurbitaceae family, have been associated with anti-obesity properties in previous studies. To determine the cucurbitacin content in CGWE, freeze-dried samples were analyzed using HPLC, revealing concentrations of approximately 137.53 μg·g^−1^ of cucurbitacin A and 1965.15 μg·g^−1^ of cucurbitacin B per dry weight sample (Figure 1A). Mice were administered CGWE for 8 weeks, and the changes in body weight and serum lipid levels were evaluated. As shown in Figure 1B, mice fed an HFD displayed a significant increase in body weight compared to the control group. However, the group treated with CGWE (HFD + CGWE) showed reduced body weight, indicating that CGWE effectively suppressed weight gain induced by the HFD. Moreover, the positive control group (HFD + orlistat) also exhibited a significant reduction in body weight. Throughout the experimental period, there were no significant differences in food intake between the groups, and the weekly food intake per mouse remained consistent among the HFD groups (Table 2). To assess the effect of CGWE treatment on lipid profiles, serum concentrations of TC, TGs, HDL, and LDL-C were analyzed. Figure 1C demonstrates that the HFD + CGWE group showed decreased levels of TC, TGs, HDL, and LDL-C compared to the HFD + vehicle group. Similarly, the HFD + orlistat group showed a significant effect on lipid profiles. These findings suggest that CGWE treatment may restore serum lipid profiles in HFD-induced obese mice, leading to a reduction in TC, TGs, HDL, and LDL-C levels. Furthermore, both CGWE and orlistat effectively suppressed HFD-induced weight gain.

### 3.2. The Inhibitory Effect of CGWE on Abdominal Fat Accumulation in HFD-Induced Obese Mice

**Figure 2:**
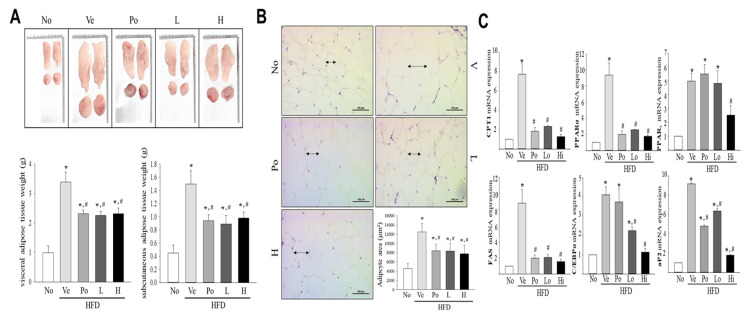
Effects of CGWE on Fat Weight and Adipocyte Size. (**A**) Abdominal fat tissues were collected from C57BL/6 mice in different treatment groups: No, HFD + vehicle, HFD + Po, or HFD + CGWE. The weights of epididymal and perirenal fat were measured. (**B**) Representative microscopic images of epididymal fat tissue sections stained with hematoxylin and eosin at a magnification of ×200. The graph shows the area of adipocytes. (**C**) Gene expression analysis of adipogenesis and lipogenesis-associated genes in liver tissue using RT-qPCR with specific primers. * *p* < 0.05 vs. the No group; # *p* < 0.05 vs. the HFD + Ve-treated group.

To explore the impact of CGWE on fat accumulation in adipose tissue, we conducted an in-depth investigation in HFD-induced obese C57BL/6 mice. After administering CGWE alongside the HFD for 8 weeks, we examined changes in abdominal fat content. The HFD + vehicle group, which received the HFD without CGWE treatment, exhibited a substantial increase in abdominal fat weight compared to the control group that consumed a normal diet. Specifically, the abdominal fat weight in the HFD + vehicle group increased by 334%, while the HFD + CGWE treatment group showed a relatively lower increase of 115% (Figure 2A). We further assessed the effect of CGWE treatment on abdominal fat accumulation using H&E staining. The HFD + vehicle group displayed significantly higher abdominal fat content compared to the untreated group. However, both the HFD + CGWE and HFD + Po groups demonstrated significant reductions in abdominal fat content when compared to the HFD + vehicle group. Similar patterns were observed in the average areas of individual adipocytes, where the HFD + CGWE and HFD + Po groups exhibited notable decreases in adipocyte area compared to the HFD + vehicle group (Figure 2B). Moreover, we analyzed the alterations in the mRNA expression levels of pivotal proteins involved in neutral lipid metabolism within the visceral adipose tissue, particularly focusing on hepatic lipid metabolism. The HFD + vehicle group showed a noteworthy increase in the mRNA expression levels of key proteins, including CPT1, PPARα, PPARγ, FAS, C/EBPα, and aP2, suggesting an increase in their corresponding protein expression levels. In contrast, the HFD + CGWE group displayed a dose-dependent decrease in the expression of these proteins. Similar significant reductions were observed in the HFD + Po-treated group, consistent with the patterns observed in the HFD + CGWE group (Figure 2C).

### 3.3. Effects of CGWE on Fat Accumulation and Lipolysis in Hepatic Tissue of HFD-Induced Obese Mice

**Figure 3:**
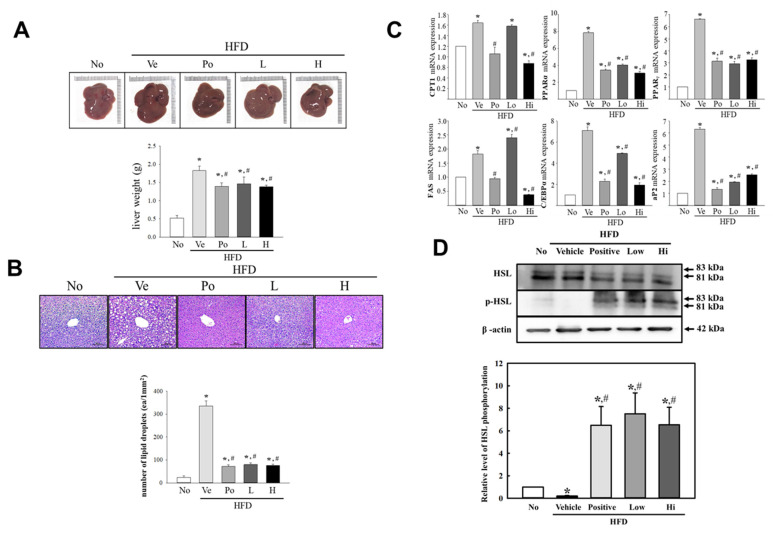
Effects of CGWE on Liver Weight, Lipid Droplet Number, Gene Expression, and Protein Phosphorylation in Liver Tissue of HFD-induced Obese Mice. (**A**) Liver weight in C57BL/6 mice treated with vehicle, Po, or CGWE. (**B**) Photomicrographs of liver tissue showing lipid droplet numbers (magnification, ×200) with quantification. (**C**) Gene expression analysis of adipogenesis and lipogenesis-associated genes in liver tissue using RT-qPCR. (**D**) Western blot analysis of lipolysis-associated proteins, including HSL. * *p* < 0.05 vs. the No group; # *p* < 0.05 vs. the HFD + ve-treated group.

Hepatic weight changes and steatosis are common features observed in obesity [4,15]. To explore CGWE’s impact on hepatic fat accumulation and lipolysis in obese mice, we conducted an 8-week study, subjecting C57BL/6 mice to a HFD while administering CGWE. We examined hepatic tissue changes to assess the effects. While there was a tendency for increased hepatic weight in the HFD + vehicle group compared to the untreated group, the difference was not statistically significant (Figure 3A). Similarly, hepatic tissue exhibited parallel patterns of lipid droplet accumulation. Significant variations in the number of lipid droplets were observed in the H&E-stained hepatic tissue sections among all groups. The HFD + vehicle group displayed a substantial increase in the number of lipid droplets, while the HFD + CGWE group showed a significant decrease (Figure 3B). To evaluate hepatic lipolytic activity, we examined changes in the mRNA expression of key proteins associated with neutral lipid metabolism, including CPT1, PPARα, PPARγ, FAS, C/EBPα, and aP2. The HFD + vehicle group demonstrated upregulation of these proteins’ mRNA expression levels. In contrast, the HFD + CGWE group showed a concentration-dependent decrease in their expression. The HFD + Po treatment also resulted in a significant decrease (Figure 3C). Furthermore, we investigated the phosphorylation of hormone-sensitive lipase (HSL) as an indicator of hepatic lipolysis. While no significant changes were observed in the untreated and HFD + vehicle groups, there was a noteworthy tendency towards a significant increase in the HFD + CGWE group (Figure 3D).

### 3.4. Attenuation of CGWE on Inflammatory Response Regulation during Hepatic Steatosis

**Figure 4:**
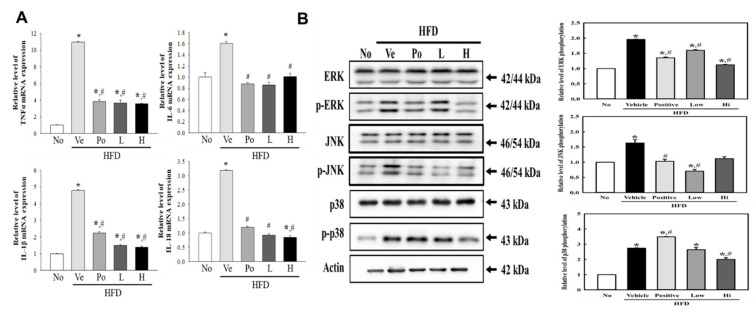
Hepatic Inflammatory Response and MAPK Signaling Pathway. (**A**) mRNA expression levels of IL-1β, IL-6, TNF-α, and NF-κB in liver tissue were analyzed using qRT-PCR. * *p* < 0.05 vs. No treatment group; # *p* < 0.05 vs. HFD + vehicle-treated group. (**B**) Western blotting analysis of p-JNK, JNK, p-ERK, ERK, p-p38, p38, and β-actin proteins in liver homogenates to assess MAPK signaling pathway activation. Representative bands shown. * *p* < 0.05 vs. the No group; # *p* < 0.05 vs. the HFD + Ve-treated group.

To investigate the impact of CGWE on inflammatory responses during hepatic steatosis, we conducted a thorough assessment of hepatic tissue. We examined changes in the expression of inflammatory mediators and the activation of the mitogen-activated protein kinase (MAPK) pathway following CGWE administration. Transcription levels of TNFα, IL-1b, IL-6, and IL-18 were significantly upregulated in the HFD + vehicle treatment group compared to the untreated group. However, both the HFD + Po and HFD + CGWE treatment groups exhibited a remarkable and statistically significant reduction in the transcription levels of these inflammatory mediators (Figure 4A). Furthermore, these changes in inflammatory mediator transcription levels were accompanied by alterations in the MAPK pathway. After HFD induction, the phosphorylation levels of ERK, JNK, and p38 were substantially higher than those in the untreated group. Nonetheless, CGWE administration led to a significant decrease in the phosphorylation of ERK, JNK, and p38, indicating its potential to dampen MAPK pathway activation (Figure 4B). These findings underscore the anti-inflammatory effects of CGWE in the context of hepatic steatosis. By modulating the expression of inflammatory mediators and mitigating the activation of the MAPK pathway, CGWE emerges as a promising therapeutic intervention for hepatic steatosis-associated inflammation.

## 4. Discussion

In the present study, we considered the effects of CGWE on fat breakdown and accumulation in HFD-induced obese rats. Excessive fat accumulation is a significant contributor to various chronic diseases, such as cancer, cardiovascular diseases, dyslipidemia, diabetes, and hypertension. Thus, the development of novel anti-obesity agents with fat-burning and fat-accumulation-inhibitory activities is of great interest. Previous studies have explored various fat-burning agents, including norepinephrine, theophylline, forskolin, isoproterenol, dibutyryl-cAMP (DBcAMP), and theophylline, which have shown potential in promoting fat breakdown in adipocytes [3,25,26]. Currently, anti-obesity drugs primarily consist of centrally acting analgesics and pancreatic lipase inhibitors like orlistat and lorcaserin. While natural remedies offer a promising avenue for isolating new active compounds to develop safe and effective anti-obesity treatments [6,27,28], limited research has been led on the anti-obesity effects of CGWE.

Our study demonstrated compelling evidence of CGWE’s effects on fat metabolism and adipose tissue. We detected a notable reduction in the mRNA levels of PPARγ, aP2, C/EBPα, and FAS in adipose tissue of HFD-induced obese rats. These results recommend that CGWE treatment may effectively suppress fat synthesis gene expression, leading to improved intracellular fatty acid metabolism. Additionally, CGWE promoted the phosphorylation of HSL, a crucial enzyme involved in neutral fat breakdown, indicating its potential to enhance lipolysis and reduce lipogenesis in obese rats. Furthermore, the anti-obesity effects of CGWE extended beyond adipose tissue. Treatment with CGWE resulted in a noteworthy reduction in overall body weight, intra-abdominal and testicular fat tissue accumulation, and adipocyte size. Moreover, CGWE-treated rats showed lower serum levels of LDL, TG, TC, and glucose compared to the control group. These findings recommend that CGWE may develop anti-obesity effects through its influence on hepatic lipid metabolism and metabolic parameters. Microscopic and histological analyses further supported the anti-obesity effects of CGWE, as evidenced by reduced fat tissue mass, decreased adipocyte size, and fewer lipid droplets. Additionally, the reduction in liver weight and lipid droplet count in the CGWE-treated group indicates its potential impact on hepatic steatosis and lipid metabolism.

Moreover, CGWE displayed anti-inflammatory properties in adipose tissue of obese rats by significantly reducing the mRNA levels of pro-inflammatory cytokines, including TNF-α, IL-1β, IL-6, and IL-18. These findings recommend that CGWE may effectively attenuate adipose tissue inflammation, which is faithfully linked to obesity-related complications such as insulin resistance and metabolic disorders. The reduction in TNF-α levels is particularly noteworthy, as TNF-α plays a significant role in obesity-related insulin resistance and metabolic dysfunction [11,29,30]. By reducing TNF-α expression, CGWE may improve insulin sensitivity and reduce lipolysis, contributing to its anti-obesity effects. Similarly, the downregulation of IL-6 mRNA levels suggests that CGWE may ameliorate the inflammatory state associated with obesity, potentially leading to improved metabolic outcomes. IL-1β and IL-18 also contribute to adipose tissue inflammation and insulin resistance, and the significant reduction in their mRNA levels following CGWE treatment indicates its potential to mitigate these inflammatory pathways and improve metabolic health in obese individuals.

Limitations of the study should be acknowledged. Although our findings provide valuable insights into the potential anti-obesity effects of CGWE, additional research is needed to elucidate the underlying molecular mechanisms and identify the specific active compounds responsible for these effects. Moreover, the study was conducted in an animal model, and extrapolation of the results to human subjects requires cautious consideration. Practical applications of the obtained results should also be addressed. The anti-obesity effects of CGWE demonstrated in this study warrant further investigation to develop potential therapeutic strategies for combating obesity and related metabolic disorders in humans. The findings highlight the importance of exploring natural remedies like CGWE as potential sources for novel anti-obesity agents. If validated in human studies, CGWE or its active components could serve as a promising natural supplement or ingredient in anti-obesity treatments and preventive strategies.

## 5. Conclusions

In this study, we investigated the effects of CGWE on fat synthesis and breakdown in adipose tissue in HFD-induced obese rats. The results revealed promising potential for CGWE as a natural anti-obesity agent. Specifically, CGWE treatment effectively reduced body weight and improved serum lipid profiles, demonstrating its ability to inhibit fat accumulation and promote fat metabolism. The observed reductions in intra-abdominal and testicular fat tissue accumulation further support CGWE’s anti-obesity properties. Moreover, CGWE exhibited anti-inflammatory effects by reducing pro-inflammatory cytokines in adipose tissue, suggesting its potential in mitigating obesity-related inflammation. Our study provides a foundation for future investigations, indicating CGWE as a safe and effective strategy for combating obesity.

## Figures and Tables

**Table 1:** Primer sequences used for the qPCR analysis.

Gene	Forward Primer (5′→3′)	Reverse Primer (3′→5′)
CPT1	GGCAGAGCAGAGGTTCAAGCT	GCCAGCGCCCGWETCAT
PPARα	TGGCAAAAGGCAAGGAGAAG	CCCTCTACATAGAACTGCAAGGTT T
PPARγ	GAGTTCATGCTTGTGAAGGATGCAAGG	CATACTCTGTGATCTCTTGCACGWE
FAS	GATCCTGGAACGWEAGAACACGWEATCTGG	AGACTGTGGAACACGWEGTGGTGGAACC
C/EBPα	GTGGACAAGAACAGCAACGAGTAC	GGAATCTCCTAGTCCTGGCTTGC
aP2	GAACCTGGAAGCTTGTCTCCAGTG	GATGCTCTTCACCTTCCTGTCGTCTGC
TNF-α	CCTGTAGCCCACGTCGTA	TTGACCTCAGCGWECTGACTTG
IL-1β	AGGCTTCCTTGTGCAAGTGT	TGAGTGACACTGCCTTCCTG
IL-6	CTCTCTGCAAGAGAGTTCCATCCAG	GCTATGGTACTCCAGAAGACCAGAGG
IL-18	GTACAAAGACAGTGAAGTAAGAGGACTG	CTCCATCTTGTTGTGTCCTGGAACACGWE
β-actin	ACGWEGCCAGGTCATCACTATT	CAAGAAGGAAGGCTGGAAAAGA

**Table 2:** Food intake of C57BL/6N mice receiving a high-fat diet for 7 weeks.

			Food Intake (g·day^−1^)				
Group	7	14	21	28	35	42	49
No	64.3 ± 5.8	63.2 ± 4.7	59.7 ± 3.8	68.5 ± 5.1	60.7 ± 5.0	67.2 ± 1.8	63.0 ± 2.3
Ve	63.0 ± 5.4	55.2 ± 6.1	63.0 ±4.3	57.8 ± 2.2	57.8 ± 1.2	56.5 ± 2.2	58.2 ± 1.7
Po	68.8 ± 5.5	62.2 ± 5.2	63.0 ± 3.8	59.7 ± 4.6	59.8 ± 4.5	63.5 ± 2.4	61.3 ± 3.0
L	63.3 ± 3.1	51.7 ± 3.9	5608 ± 3.0	57.3 ± 3.4	54.7 ± 2.8	50.5 ± 3.7	48.2 ± 3.4
H	62.8 ± 2.8	50.5 ± 2.0	60.7 ± 3.4	54.4 ± 2.9	53.5 ± 2.6	53.0 ± 4.4	52.3 ± 3.2

Food intake was compared among the experimental groups. The percentage body weight gain from 0 to 7 weeks was calculated. HFD + vehicle-treated group.

## Data Availability

The data presented in this study are available on request from the corresponding author.

## References

[1] Aldamarany W.A.S., Taocui H., Liling D., Mei H., Yi Z., Zhong G. (2023). Perilla, sunflower, and tea seed oils as potential dietary supplements with anti-obesity effects by modulating the gut microbiota composition in mice fed a high-fat diet. Eur. J. Nutr..

[2] Gopal S.S., Sukhdeo S.V., Vallikannan B., Ponesakki G. (2023). Lutein ameliorates high-fat diet-induced obesity, fatty liver, and glucose intolerance in C57BL/6J mice. Phytother. Res..

[3] Kim W., Kwon H.J., Jung H.Y., Lim S., Kang B., Jo Y., Yu D., Choi S.Y., Hwang I.K., Kim D.W. (2021). Extracts from the leaves of *Cissus verticillata* ameliorate high-fat diet-Induced memory deficits in mice. Plants.

[4] Moon J., Ha M.J., Shin M., Kim O.Y., Yoo E.H., Song J., Chung J.H. (2019). *Semen cuscutae* administration improves hepatic lipid metabolism and adiposity in high fat diet-induced obese mice. Nutrients.

[5] Li D., Xu Z., Li Y., Gan L., Wu P., Wu R., Jin J., Zheng X., Zhang K., Ma H. (2022). Polysaccharides from *Callerya speciosa* alleviate metabolic disorders and gut microbiota dysbiosis in diet-induced obese C57BL/6 mice. Food Funct..

[6] Yuan D., Huang Q., Li C., Fu X. (2022). A polysaccharide from *Sargassum pallidum* reduces obesity in high-fat diet-induced obese mice by modulating glycolipid metabolism. Food Funct..

[7] Lan Y., Sun Q., Ma Z., Peng J., Zhang M., Wang C., Zhang X., Yan X., Chang L., Hou X. (2022). Seabuckthorn polysaccharide ameliorates high-fat diet-induced obesity by gut microbiota-SCFAs-liver axis. Food Funct..

[8] Wang J., Zhang Y., Shen Q., Wu J., Li J. (2021). Oleanolic acid derivative HA-20 inhibits adipogenesis in a manner involving PPARγ-FABP4/aP2 pathway. J. Mol. Endocrinol..

[9] Han J., Kim M., Myung C. (2022). *Garcinia cambogia* improves high-fat diet-induced glucose imbalance by enhancing calcium/CaMKII/AMPK/GLUT4-mediated glucose uptake in skeletal muscle. Mol. Nutr. Food Res..

[10] Lee J., Kim T.Y., Kang H., Oh J., Park J.W., Kim S., Kim M., Apostolidis E., Kim Y., Kwon Y. (2021). Anti-obesity and anti-adipogenic effects of chitosan oligosaccharide (GO2KA1) in SD rats and in 3T3-L1 preadipocytes models. Molecules.

[11] Allam E.A., Ibrahim H.F., Abdulmalek S.A., Abdelmeniem I.M., Basta M. (2022). Coenzyme Q(10) alleviates testicular endocrine and spermatogenic dysfunction induced by high-fat diet in male Wistar rats: Role of adipokines, oxidative stress and MAPK/ERK/JNK pathway. Andrologia.

[12] Inamdar S., Joshi A., Malik S., Boppana R., Ghaskadbi S. (2019). Vitexin alleviates non-alcoholic fatty liver disease by activating AMPK in high fat diet fed mice. Biochem. Biophys. Res. Commun..

[13] Wang K., Liang C., Cao W., Luo G., Zhong S., Zeng Z., Dai L., Song J. (2022). Dietary sinapic acid attenuated high-fat diet-induced lipid metabolism and oxidative stress in male Syrian hamsters. J. Food Biochem..

[14] Choi R., Lee M. (2021). *Polygonum multiflorum* Thunb. hot water extract reverses high-fat diet-induced lipid metabolism of white and brown adipose tissues in obese mice. Plants.

[15] Yin K., Zhou X., Jiang W., Wang L., Dai Z., Tang B. (2021). Jiangzhi ligan decoction inhibits GSDMD-mediated canonical/noncanonical pyroptosis pathways and alleviates high-fat diet-Induced nonalcoholic fatty liver disease. Dis. Markers.

[16] Sebastian P., Schaefer H., Telford I.R.H., Renner S.S. (2010). Cucumber (*Cucumis sativus*) and melon (*C. melo*) have numerous wild relatives in Asia and Australia, and the sister species of melon is from Australia. Proc. Natl. Acad. Sci. USA.

[17] Villanueva M.J., Tenorio M.D., Esteban M.A., Mendoza M.C. (2004). Compositional changes during ripening of two cultivars of muskmelon fruits. Food Chem..

[18] Mallek-Ayadi S., Bahloul N., Baklouti S., Kechaou N. (2022). Bioactive compounds from *Cucumis melo* L. fruits as potential nutraceutical food ingredients and juice processing using membrane technology. Food Sci. Nutr..

[19] Kim H., Kang Y. (2010). Antioxidant activity of ethanol extracts of non-edible parts (stalk, stem. leaf, seed) from oriental melon. Korean J. Plant Resour..

[20] Silva M.A., Albuquerque T.G., Alves R.C., Oliveira M.B.P., Costa H.S. (2020). Melon (*Cucumis melo* L.) by-products: Potential food ingredients for novel functional foods?. Trends Food Sci Technol..

[21] Guo L., Park S.Y., Kang H.M., Kang N.J., Hwang D.Y., Choi Y. (2022). Edible Vitalmelon fruit extract inhibits adipogenesis and ameliorates high-fat diet-induced obesity. BioMed Res. Int..

[22] Kim Y., Choi D., Cha A., Lee Y., Baek N., Rimal S., Sang J., Lee Y., Lee S. (2020). Critical enzymes for biosynthesis of cucurbitacin derivatives in watermelon and their biological significance. Commun. Biol..

[23] Aderiye B.I., David O.M., Fagbohun E.D., Faleye J., Olajide O.M. (2020). Immunomodulatory and phytomedicinal properties of watermelon juice and pulp (*Citrullus lanatus* Linn): A review. GSC Biol. Pharm. Sci..

[24] Maja D., Mavengahama S., Mashilo J. (2022). Cucurbitacin biosynthesis in cucurbit crops, their pharmaceutical value and agricultural application for management of biotic and abiotic stress: A review. S. Afr. J. Bot..

[25] Kim E.A., Yang J., Byeon E., Kim W., Kang D., Han J., Hong S., Kim D., Park S., Huh J. (2021). Anti-obesity effect of pine needle extract on high-fat diet-induced obese mice. Plants.

[26] Lee Y., Kim H., Nam S., Chu J., Kim J., Lim J., Kim S., Sung M. (2022). Protective effects of high-fat diet against murine colitis in association with leptin signaling and gut microbiome. Life.

[27] Lindfors S., Polianskyte-Prause Z., Bouslama R., Lehtonen E., Mannerla M., Nisen H., Tienari J., Salmenkari H., Forsgård R., Mirtti T. (2021). Adiponectin receptor agonist AdipoRon ameliorates renal inflammation in diet-induced obese mice and endotoxin-treated human glomeruli ex vivo. Diabetologia.

[28] Saber S., Abd El-Fattah E.E., Yahya G., Gobba N.A., Maghmomeh A.O., Khodir A.E., Mourad A.A.E., Saad A.S., Mohammed H.G., Nouh N.A. (2021). A novel combination therapy using rosuvastatin and lactobacillus combats dextran sodium sulfate-induced colitis in high-fat diet-fed rats by targeting the TXNIP/NLRP3 interaction and influencing gut microbiome composition. Pharmaceuticals.

[29] Zhang X., Nie Q., Zhang Z., Zhao J., Zhang F., Wang C., Wang X., Song G. (2021). Resveratrol affects the expression of uric acid transporter by improving inflammation. Mol. Med. Rep..

[30] Ge C., Xu M., Qin Y., Gu T., Lou D., Li Q., Hu L., Nie X., Wang M., Tan J. (2019). Fisetin supplementation prevents high fat diet-induced diabetic nephropathy by repressing insulin resistance and RIP3-regulated inflammation. Food Funct..

